# Disruptions, distractions, and discoveries: Doctoral students’ reflections on a pandemic

**DOI:** 10.1177/1473325020973341

**Published:** 2021-03

**Authors:** Chinyere Y Eigege, Priscilla P Kennedy

**Affiliations:** Graduate College of Social Work, University of Houston, Houston, USA

**Keywords:** Crisis, collaboration, reflexivity, social justice, social work education, health

## Abstract

This paper describes the reflections of two social work PhD students based on their personal and professional experiences with the COVID-19 pandemic. The students describe their positionality and use that to expound on the impact of the pandemic on their lives. They reflect on the disruptions to their social work education and research priorities including transitioning to online learning and modifications to research agendas. They then discuss ongoing distractions such as worries about getting sick, mental health concerns, and financial constraints. They share their discoveries about glaring disparities in coronavirus infection and death rates, the need to adjust research agendas in response to current events, and the urgency for qualitative research strategies to add meaning to the numbers being reported. In addition, the authors describe shared experiences and intersections they discovered while writing this essay. Finally, recommendations for practice include recommitting to social work values to help surmount the ongoing waves of this pandemic; reimagining social work education so that disparities and injustice intersect with every subject taught and graduates become experts at leading social change; and harnessing the untapped potential of qualitative research to drive real, systemic change.

The beginning of this year was the start of a new decade and held the promise of concluding another year of doctoral education while getting closer to a PhD in social work. This was supposed to be the year of refining research agendas and honing knowledge and skills for a qualitative research career. Everything was on track until the coronavirus made its way into the United States. The disruptions that ensued were unprecedented. From shutdowns to social distancing to sanitary practices, our world came to a grinding halt in an effort to slow the spread.

With these disruptions came adjustments to a new way of life. Home was designated the only safe place for all things – living, homeschooling, and working. At the same time, unanticipated distractions crept in: concerns about getting sick or passing the virus along to loved ones; worries about mental health for self and family members; and tighter financial constraints with furloughs and job losses. Life was lived in “survival mode,” trying to safely get from one place to the next and from one day to the next. Many of our plans and goals were scrapped or whittled down to accommodate the realities of living in a pandemic.

As we began watching and listening to the experiences of others, it became apparent that although we were all experiencing the same event – COVID-19 – we were not all impacted in the same way. The glaring disparities in infection rates, death rates, unemployment rates, and food and housing insecurity revealed the pre-existing condition of social injustice and inequities in our society. Although we were not naïve to this fact, it was in sharing and listening to each other that we discovered the blind spots in our reflections and reflexivity about our own and each other’s racial identities, our doctoral education thus far, and our mutual interest in qualitative research. Below are our personal and shared stories, highlighting the disruptions, distractions, and discoveries experienced by two doctoral students during the COVID-19 pandemic.

## Doctoral students’ reflections

### Chinyere

I am a fourth-year doctoral student using qualitative research methods to improve maternal health outcomes for Black women. I identify as a Black woman and a mother. I am a licensed clinical social worker practicing full time while concurrently working towards my PhD in social work. Although this is financially necessary for my young family, it has come at a cost during my doctoral education, as I am limited to certain resources and educational opportunities due to my part-time status. In the time that I have been learning and training to become a qualitative health researcher, I am constantly reminded of the disparities that exist for communities of color in every social and medical issue.

Discussing these disparities as we have studied them, but also having seen them first-hand has been a defining feature of my relationship with Priscilla. She is a fellow doctoral student whose reflection is shared below. Even when we met during our master’s program, her passion for addressing systemic issues of racial injustice was evident. Now, as my colleague in the doctoral program, she is fueled by her experiences working to address health disparities for the Black and Hispanic residents in the OST- South Union neighborhood where I live. Her willingness to listen to me and my perspective as an equally passionate insider has nurtured a safety and trust in our relationship that allowed us to have some honest reflections and dialogue about the ways our lives have been differently impacted by COVID-19.

A few days into spring break, it became clear as I received notices from my kids’ schools and my PhD program that school would continue virtually after adding another week to the break to prepare for online learning. As a doctoral student, this was not that big of a leap in learning. The main change that I did not anticipate was having to continue my academic engagement, my full-time job, and manage my children’s academics concurrently, while trying to keep us all safe. Additionally, I was not prepared for how much COVID-19 would change the way I thought about and engaged with my doctoral research.

Qualitative social science research is born out of inquiry, a gap in knowledge, a quest for deeper understanding and meaning, and a need to amplify underrepresented voices and perspectives. As we continue to adjust to life during this pandemic, the flaws in social systems have been exposed, revealing areas that require immediate responses. Racial disparities in infection and death rates are not just statistics anymore, as I live in one of the neighborhoods hardest hit because of its high population of low-income Black and Brown residents. As a burgeoning qualitative researcher, the current reality has reshaped the way I engage with my education and research.

Impossible to ignore as a Black woman, partnered to a Black man, raising Black children, doing life with my Black neighbors, were the emotions surrounding the concurrent pandemic of racism that was highlighted during this time. In addition to trying to keep me and my family safe from the coronavirus, I was also tasked with managing mine and my family’s fears and anxieties related to racism and the unjust killings of Black people in America. Although I am a practicing clinical social worker, I was not prepared for the mental toll the convergence of these events would have on me, my partner, my children, my neighbors, and my clients. The constant and recurring trauma of images, video clips, and phrases were triggering and emotionally draining as I carried the burden of fear with very few safe spaces to share my worries.

Born out of this pandemic are research questions and agendas that are not entirely new but have now been put in the spotlight. Now more than ever, there is a sense of urgency to expose the racial divide in the ways that Black individuals and communities are navigating our current realities. COVID-19 infection and death rates are about twice as high among Black individuals compared to White individuals (Centers for Disease Control and Prevention [CDC], 2020). However, little is being said about the mental toll that COVID-19 has brought on Black communities. With schools closed, many families that depend on school meals are now facing food insecurity. Essential workers in our service industries and the support workers for most of our facilities that need to stay open, such as hospitals, struggle with the tension of risking exposure for themselves and their families or going into work. These individuals do not have the luxury of working safely from home.

As we continue to adapt to an evolving world with the effects of a deadly virus, and a society that is now awakened to the racism that infects many aspects of life for Black and Brown communities, I am convicted in the urgency of prioritizing education and research related to these issues. Qualitative research strategies are best poised to engage people in these difficult circumstances, elevating their voices and narratives so we can truly understand the meaning behind the numbers we see. As a qualitative researcher who identifies as an insider to many of the issues that I will be researching, it is important I continue to seek and foster safe and trusted spaces to design, process, and analyze my research. I am thankful for the faculty and colleagues of color that have chosen to commit to this work. Additionally, I am encouraged by colleagues like Priscilla, who are not insiders experiencing the racial injustices they study but are committed to do the hard work of listening to, learning from, and advocating for communities like mine.

### Priscilla

I am a second-year doctoral student who is also a middle-aged White woman. I plan to conduct qualitative inquiry that seeks to understand how historical and cultural factors shape and impact the way people experience racial residential segregation. Writing this reflection has compelled me to put into words the advantages that my white identity affords me. I have the ability to attend school full-time without working; to stay at home during the COVID-19 pandemic; to access health care that covers COVID-19 testing for me and my family; to send my children to college without going into debt; and to lead the type of life where I can research, write, and speak about racial inequality without ever having lived it.

Chinyere and I met in 2013 when we were assigned to the same cohort of the MSW program at the University of Houston (UH) in Houston, Texas. From day one, I was inspired by her incisive contributions to class discussions. I was also afraid she could see through my utter lack of consciousness about how the mechanisms of white supremacy and systemic racism have privileged my life. Beyond what I learned in class lectures, textbooks, and internships, my friendship with Chinyere turned my worldview upside down. Without judging me or rejecting me, she gave me space to struggle with the hard truth that shrugging off my racial identity did not give me a pass; it made me passively complicit. The hard truth I own is that the White race – my race – is responsible for building a system of White dominance designed specifically to deny equality for Black and Brown people. And I have greatly benefitted from that system.

Similar to Chinyere’s experience, I was fortunate that disruptions in my educational trajectory due to the pandemic were mitigated by faculty at UH. If there is a silver lining to the distractions caused by coronavirus, it is that I was given a rare opportunity to immerse myself in exigent research on the mental health impacts of COVID-19.

Among the best laid plans, mine included an independent study of qualitative inquiry in the context of racial residential segregation with my mentor. However, our work scarcely got off the ground before it was disrupted by the coronavirus lockdown in Houston. At the same time our campus was closed, I felt myself powering down. The urge to turn off the madness and retreat was overwhelming, but I still had papers to write, presentations to give, statistics homework to finish, and teenage children to wrangle. The work I produced was mediocre at best. I learned to compartmentalize and focus on positive experiences, such as reconnecting with my mentor to revisit the goals and objectives of my independent study.

With our discussions centered on the serious, long-term mental health impacts of COVID-19 on the most vulnerable, my mentor and I pivoted to more urgent and relevant research. We searched the literature, discussed findings and gaps in knowledge, and discovered a critical thread: the necessity of gathering stories to document the challenges Black women uniquely face during the pandemic due to overlapping systems of oppression. As a fledgling qualitative researcher, I came to understand the imperative of capturing stories of lived experiences so we can respond in a way that honors people’s histories instead of erasing them.

As an outsider, I anticipated it would be uncomfortable for me to talk about the corrupt systems and racist policies created by White people that facilitated the disproportionate sickness and death of Black Americans from COVID-19. I discovered that being an outsider meant I took nothing for granted, so I constantly asked questions to clarify my understanding. My mentor set a place for me in our research, prompted me to examine my own lived experiences in a White-centered world, and helped me discover groundbreaking work by researchers, practitioners, and activists such as Kimberlé Crenshaw, Derrick Bell, Mary Pattillo-McCoy, Samuel Aymer, and Richard Rothstein. I feel fortunate that in spite of the disruptions and distractions of my PhD education due to coronavirus, I am becoming a more conscious social work researcher by deepening my knowledge base and positioning myself for my future research.

Collaborating with Chinyere in this season has revealed for me the stark differences between us in terms of our racial identities, cultures, and work/life experiences. Yet we intersect in one important way: our shared struggle to conjure reflexivity when what we really want to do is shut down emotionally, hunker down, and focus on protecting our families. The idea of being vulnerable and open to self-examination, when at the same time our basic needs for safety and security are threatened, feels extravagant and a little dangerous. As we slowly leaned into each other during this process, Chinyere and I rediscovered our easy rapport and complementary personalities. Our continued engagement in this reflexive process has been a time of growth and challenge, leading to greater consciousness in my positionality as a qualitive researcher. An important goal for my future qualitative research is to position myself as (a) someone who is conscious that I live in a White-centered world, which greatly advantages me; and (b) someone who has learned to follow, listen to, and learn from people in Black and Brown communities.

## Conclusion

During this time where there is so much division, especially around racial disparities, we are encouraged to see how much our reflexivity has moved us to thinking about these issues on a level beyond our personal reflections and experiences. The core values of social work oblige us to not only recognize these issues, but to actively engage them in our work addressing health disparities among Black women and the impacts of racial residential segregation.

The reflexive work we continue to do in our relationship has impacted the quality of our doctoral scholarship and can be a model that proves differences are not a threat to progress or to finding solutions. Our missteps and growth through this process are a testament to the complementary ways of doing this research. In fact, we believe that insider-outsider collaboration may be key to enhancing the depths and insights that can be reached through qualitative research.

As our world adapts to this new normal, social work practice and research need to adjust as well. Just as telemedicine has now been elevated to provide access to mental health services, social work research needs to embrace this opportunity. The narratives and experiences of people living through this moment in time need to be captured and supported. For too long they have been sidelined. Qualitative research and the model of collaboration we are practicing need institutional support through curricula, funding, and publications.

Emerging social work researchers need to be encouraged to explore non-traditional modes of research, as the conventional methods are no longer sufficient to capture the impact of the times in which we are living. Numbers alone cannot tell the full story. Social work has the opportunity to lead in the training and scholarship of a new breed of researchers who are skilled in conducting research in the ways we need it done now. Let us reimagine social work education so that future graduates like us can become experts at leading sweeping and intentional social change.
